# Ciliary signaling proteins are mislocalized in the brains of Bardet-Biedl syndrome 1-null mice

**DOI:** 10.3389/fcell.2022.1092161

**Published:** 2023-01-09

**Authors:** Toneisha Stubbs, James I. Bingman, Jason Besse, Kirk Mykytyn

**Affiliations:** Department of Biological Chemistry and Pharmacology, College of Medicine, The Ohio State University, Columbus, OH, United States

**Keywords:** cilia, Bardet-Biedl syndrome, ciliopathy, Gpr161, GPR19, beta arrestin

## Abstract

In the brain, primary cilia are found on most, if not all, central neurons. The importance of neuronal cilia is underscored by the fact that human diseases caused by primary cilia dysfunction, which are known as ciliopathies, are associated with neuropathologies, including neuropsychiatric disorders and learning and memory deficits. Neuronal cilia are enriched for certain G protein-coupled receptors and their downstream effectors, suggesting they sense and respond to neuromodulators in the extracellular milieu. GPCR ciliary localization is disrupted in neurons from mouse models of the ciliopathy Bardet-Biedl syndrome, with GPCRs failing to localize to cilia, indicating the Bardet-Biedl syndrome proteins are required for trafficking of G protein-coupled receptors into neuronal cilia. Yet, dopamine receptor 1 accumulates in cilia in the absence of Bardet-Biedl syndrome proteins, suggesting Bardet-Biedl syndrome proteins are required for normal ciliary import and export. To further explore the roles of the Bardet-Biedl syndrome proteins in neuronal cilia, we examined localization of ciliary signaling proteins in a new constitutive Bbs1 knockout mouse model. Interestingly, we find that two additional ciliary G protein-coupled receptors (Gpr161 and Gpr19) abnormally accumulate in cilia on Bardet-Biedl syndrome neurons. In addition, we find that the GPCR signaling protein β-arrestin accumulates in a subset of cilia in the brain, suggesting the presence of additional unidentified ciliary G protein-coupled receptors. These results confirm the importance of the Bardet-Biedl syndrome proteins in establishing ciliary GPCR pathways and indicate that loss of Bbs1 leads to complex changes in the localization of signaling proteins in the brain.

## Introduction

Primary cilia are signaling organelles that mediate specialized signal transduction pathways critical for normal development and cellular homeostasis (Anvarian et al., 2019). These pathways are established and maintained by coordinating the trafficking of proteins into and out of cilia (Nachury and Mick, 2019). The Sonic hedgehog (Shh) pathway is a well characterized example of the functional importance of dynamic localization of ciliary signaling components. In the absence of Shh, the Shh receptor Patched (Ptch) and orphan GPCR Gpr161 are enriched in cilia and maintain repression of the pathway (Rohatgi et al., 2007; Mukhopadhyay et al., 2013). Shh binding results in Ptch and Gpr161 removal from cilia and concomitant ciliary accumulation of the GPCR-like Smoothened (Smo), which is the major transducer of Shh signaling, leading to pathway activation (Corbit et al., 2005; Rohatgi et al., 2007). β-arrestins, scaffolding proteins that regulate GPCR signaling and internalization, are recruited to activated Gpr161 and mediate its export from cilia (Pal et al., 2016). Overall, the dynamic localization of Shh signaling components to cilia is critical for proper Shh signaling and defects in Shh signaling are associated with ciliopathies.

Ciliopathies are associated with neuropathologies, such as hyperphagia-induced obesity, neuropsychiatric disorders, and learning and memory deficits (Suciu and Caspary, 2021). Certain GPCRs are enriched in neuronal cilia, including somatostatin receptor 3 (Sstr3) (Handel et al., 1999), serotonin receptor 6 (Htr6) (Brailov et al., 2000), melanin-concentrating hormone receptor 1 (Mchr1) (Berbari et al., 2008a), dopamine receptor 1 (D1) (Domire et al., 2011), Neuropeptide Y receptor 2 (NPY2) (Loktev and Jackson, 2013), kisspeptin receptor 1 (Kiss1r) (Koemeter-Cox et al., 2014), and melanocortin four receptor (MC4R) (Siljee et al., 2018). Neuronal cilia also contain the GPCR effectors type 3 adenylyl cyclase (AC3) (Bishop et al., 2007) and β-arrestin (Green et al., 2016), suggesting that GPCRs signal on the ciliary membrane. Indeed, numerous studies have now shown that GPCR ciliary localization is critical for normal signaling. Ablation of cilia on Kiss1r-positive neurons reduces agonist-mediated increases in firing rate in the mouse hypothalamus (Koemeter-Cox et al., 2014). Disruption of Sstr3 ciliary signaling results in increased neuronal excitability in rat neocortical neurons (Tereshko et al., 2021). Disruption of ciliary Mchr1 leads to decreased agonist-mediated cAMP and extracellular signal-regulated kinase signaling in mouse hypothalamic neurons (Hsiao et al., 2021). Inhibition of MC4R ciliary signaling in the mouse hypothalamus results in hyperphagia-induced obesity (Wang et al., 2021). Mice with disrupted D1 ciliary localization have a reduction in D1-mediated signaling in the striatum that is associated with a decrease in locomotor activity and obesity (Stubbs et al., 2022). Recently, synapses between serotonergic axons and primary cilia of hippocampal CA1 neurons have been described in the mouse brain (Sheu et al., 2022). Importantly, serotonin release stimulates ciliary Htr6 and activates a non-canonical signaling pathway to increase chromatin accessibility (Sheu et al., 2022). Overall, ciliary GPCR signaling appears to be important for normal neuronal function.

GPCR localization to neuronal cilia is dynamic and regulated by environmental cues. For example, D1 ciliary localization is increased in response to an increase in cyclic AMP levels and decreased in response to agonist treatment (Domire et al., 2011). NPY2 and Sstr3 are also exported from neuronal cilia in response to agonist (Loktev and Jackson, 2013; Green et al., 2016). The proteins disrupted in the ciliopathy Bardet-Biedl syndrome (BBS) are important regulators of ciliary localization of GPCRs in the brain. BBS is a heterogeneous autosomal recessive disorder characterized by a wide range of phenotypes, including obesity, behavioral disturbances, and cognitive deficits (Forsythe and Beales, 2013). A group of BBS proteins (BBS1, 2, 4, 5, 7, 8, 9, and 18) form a complex called the BBSome (Nachury et al., 2007; Loktev et al., 2008). The BBSome is required for ciliary localization of Sstr3, Mchr1, and NPY2 (Berbari et al., 2008b; Loktev and Jackson, 2013). Yet, D1 accumulates in neuronal cilia in BBSome mutant mice (Domire et al., 2011; Zhang et al., 2013) and does not undergo agonist-mediated ciliary export on BBSome mutant neurons (Domire et al., 2011). Taken together, these results suggest that the BBSome is required for trafficking of some GPCRs into cilia and others out of cilia in the brain.

Previous studies characterizing the impact of Bbs1 loss in mice utilized a model where the most common human BBS mutation (M390R) was knocked into the mouse gene (Davis et al., 2007). Although M390R knock-in mice exhibit phenotypes consistent with mice lacking a BBSome subunit, we decided to generate a constitutive Bbs1 knockout mouse model, as loss of Bbs1 protein is known to disrupt BBSome formation and function (Guo et al., 2016). We then performed an analysis of the consequences of Bbs1 loss on the ciliary localization of specific signaling proteins in the brain.

## Results

### Loss of Bbs1 in the mouse brain impacts GPCR ciliary localization

**FIGURE 1:**
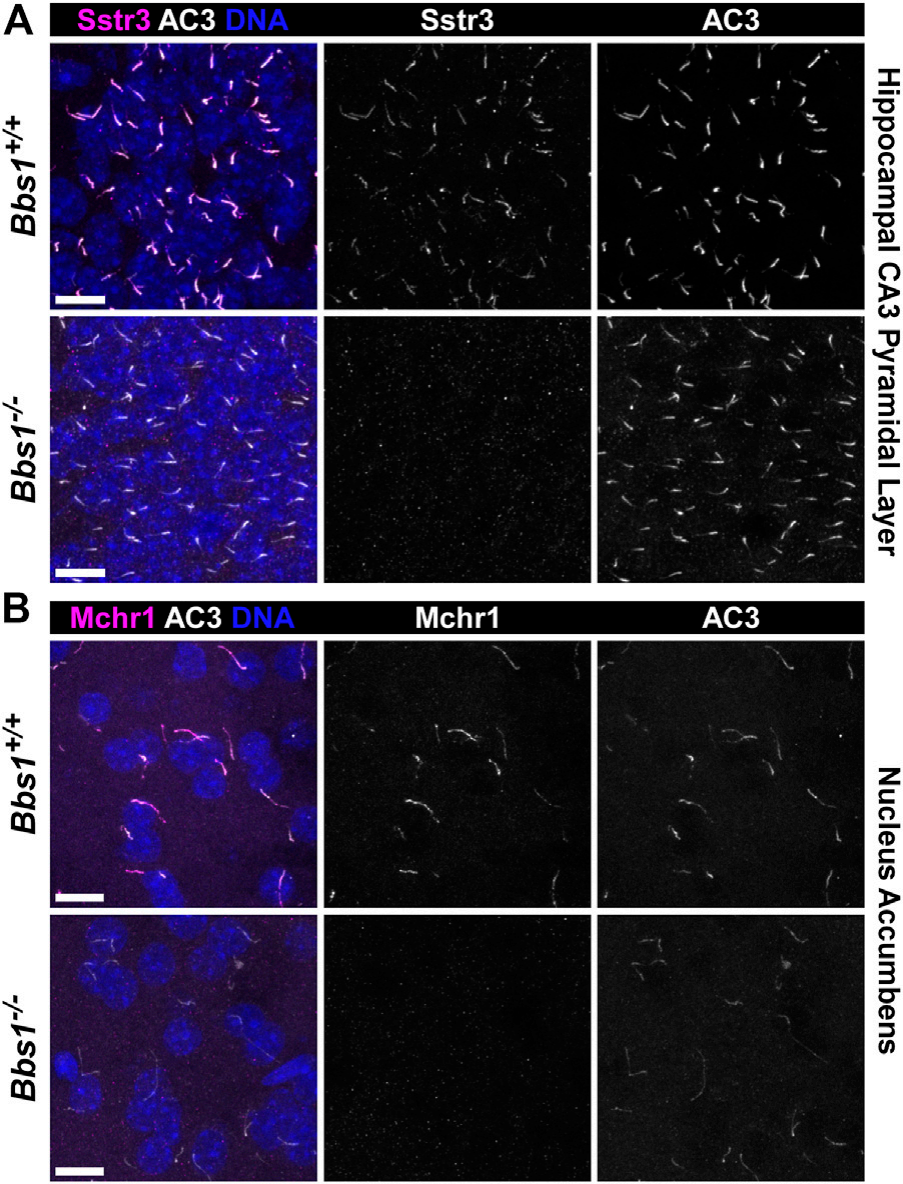
*Bbs1* ^*−/−*^ mice lack Sstr3 and Mchr1 ciliary localization in the brain **(A)** Representative images of the pyramidal layer of the hippocampal CA3 region from adult *Bbs1* ^*+/+*^ (upper row) and *Bbs1* ^*−/−*^ (lower row) mice colabeled for Sstr3 (magenta) and AC3 (white). Sstr3 localized to 74% (623/846; *n* = 3 animals) of AC3-positive cilia in *Bbs1* ^*+/+*^ sections and 0% (0/417; *n* = 3 animals) of AC3-positive cilia in *Bbs1* ^*−/−*^ sections **(B)** Representative images of the nucleus accumbens from adult *Bbs1* ^*+/+*^ (upper row) and *Bbs1* ^*−/−*^ (lower row) mice colabeled for Mchr1 (magenta) and AC3 (white). Mchr1 localized to 72% (675/938; *n* = 3 animals) of AC3-positive cilia in *Bbs1* ^*+/+*^ sections and 0% (0/471; *n* = 3 animals) of AC3-positive cilia in *Bbs1* ^*−/−*^ sections. Nuclei are stained with DRAQ5 (blue). Scale bars represent 10 µm.

The conditional Bbs1 knockout allele has loxP sites flanking exon three and *Bbs1* mRNA is lost following recombination (Carter et al., 2012). We crossed conditional Bbs1 knockout mice with transgenic mice expressing Cre recombinase under control of the CMV promoter, which is expressed in all tissues, in order to generate mice carrying a germline deletion of the Bbs1 allele. We then intercrossed mice heterozygous for a wildtype and null allele (*Bbs1* ^*+/−*^) to generate wildtype (*Bbs1* ^*+/+*^) and constitutive knockout (*Bbs1* ^*−/−*^) animals. To test whether loss of Bbs1 impacts ciliary GPCR localization in the brain, we colabeled brain sections from adult *Bbs1* ^*+/+*^ and *Bbs1* ^*−/−*^ mice with antibodies against Sstr3, Mchr1, or D1 and an antibody against AC3, which serves as a marker for neuronal cilia (Bishop et al., 2007). In *Bbs1* ^*+/+*^ sections we detected ciliary localization of both Sstr3 and Mchr1 in numerous brain regions, with especially abundant labeling in the CA3 region of the hippocampus and nucleus accumbens, respectively (Figures 1A, B). Similar to other BBSome mutant mice (Berbari et al., 2008b), we failed to detect ciliary localization of either Sstr3 or Mchr1in brain sections from *Bbs1* ^*−/−*^ mice (Figures 1A, B). The lengths of AC3-positive cilia were similar between both genotypes, indicating that loss of ciliary localization is not due to overt ciliary defects in *Bbs1* ^*−/−*^ mice (Table 1).

**TABLE 1:** Summary of GPCR ciliary localization and ciliary length in *Bbs1* ^*+/+*^ and *Bbs1* ^*−/−*^ brain regions.

Region	GPCR	*Bbs1* ^*+/+*^ localization	*Bbs1* ^*−/−*^localization	Cilia lengths (µm)^a^
CA3 region hippocampus^b^	Sstr3	Y	N	*Bbs1* ^*+/+*^: 4.203 ± 0.08123
				*Bbs1* ^*−/−*^: 3.991 ± 0.1034
				Not significantly different *p* = 0.1060
Nucleus accumbens^c^	Mchr1	Y	N	*Bbs1* ^*+/+*^: 5.825 ± 0.1160
				*Bbs1* ^*−/−*^: 5.995 ± 0.1808
				Not significantly different *p* = 0.4069
Striatum^d^	D1	N	Y	*Bbs1* ^*+/+*^: 6.625 ± 0.1931
				*Bbs1* ^*−/−*^: 7.179 ± 0.3075
				Not significantly different *p* = 0.1243
Ventral CA1 region^e^	Gpr161	Y	Y	*Bbs1* ^*+/+*^: 4.520 ± 0.1151
				*Bbs1* ^*−/−*^: 5.424 ± 0.2135
				Significantly different *p* < 0.0001

^a^ Lengths recorded as mean (µm) ± S.E.M.

^b^ In the CA3 region of the hippocampus *n* = 390 *Bbs1* ^*+/+*^ cilia and *n* = 379 *Bbs1* ^*−/−*^ cilia.

^c^ In the nucleus accumbens *n* = 390 *Bbs1* ^*+/+*^ cilia and *n* = 237 *Bbs1* ^*−/−*^ cilia.

^d^ In the striatum *n* = 135 *Bbs1* ^*+/+*^ cilia and *n* = 128 *Bbs1* ^*−/−*^ cilia.

^e^ In the ventral CA1 region of the hippocampus *n* = 258 *Bbs1* ^*+/+*^ cilia and *n* = 190 *Bbs1* ^*−/−*^ cilia.

**FIGURE 2:**
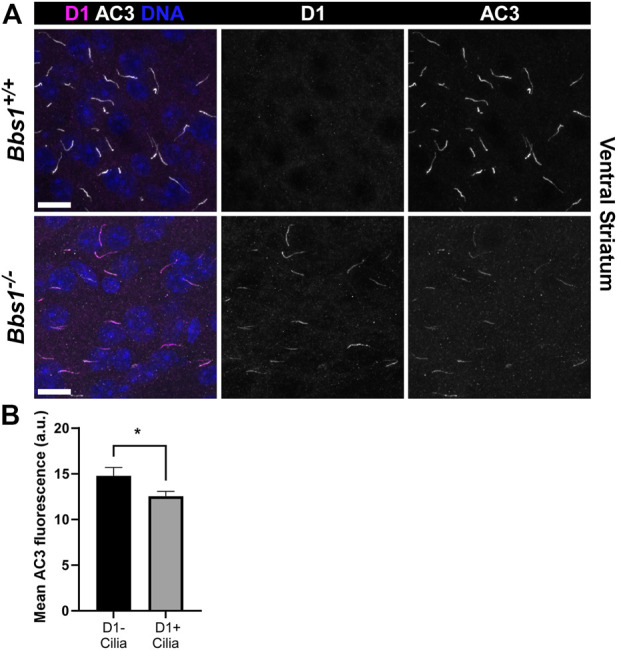
D1 accumulates in cilia in the brains of *Bbs1* ^*−/−*^ mice and is associated with a reduction in AC3 ciliary signal **(A)** Representative images of the ventral striatum from adult *Bbs1* ^*+/+*^ (upper row) and *Bbs1* ^*−/−*^ (lower row) mice colabeled for D1 (magenta) and AC3 (white). D1 localized to 0% (0/739; *n* = 3 animals) of AC3-positive cilia in *Bbs1* ^*+/+*^ sections and 64% (279/434; *n* = 3 animals) of AC3-positive cilia in *Bbs1* ^*−/−*^ sections. Nuclei are stained with DRAQ5 (blue). Scale bars represent 10 µm **(B)** Quantification of mean ciliary AC3 fluorescence in D1-negative (*n* = 100) and D1-positive (*n* = 194) cilia in *Bbs1* ^*−/−*^ sections. Note that the AC3 signal is significantly lower in D1-positive cilia. Values are expressed as mean ± SEM. *p < 0.05.

D1 ciliary localization was not detected in *Bbs1* ^*+/+*^ sections. However, in *Bbs1* ^*−/−*^ sections we observed abundant D1 ciliary localization in several brain regions, including the striatum, amygdala, and olfactory tubercle (Figure 2A), similar to other BBSome mutant mice. Interestingly, we consistently observed a reduction in AC3 labeling in cilia in the striatum of *Bbs1* ^*−/−*^ mice compared to *Bbs1* ^*+/+*^ mice. Quantification of mean ciliary AC3 fluorescence in D1-negative and D1-positive cilia in the striatum of *Bbs1* ^*−/−*^ sections confirmed that AC3 signal is reduced in cilia that are enriched for D1 (t = 2.271, df = 286, *p* < 0.05) (Figure 2B). The lengths of AC3-positive cilia were similar between both genotypes (Table 1), indicating that the reduction in AC3 fluorescence is not due to differences in cilia length. We previously reported the same observation after conditionally disrupting Bbs1 in D1-expressing neurons and proposed that the ciliary accumulation of D1 may be preventing AC3 ciliary localization or interfering with binding of the AC3 antibody (Stubbs et al., 2022). Nevertheless, together these results are consistent with findings in other BBSome mutant mice and suggest that the BBSome is required for ciliary entry or retention of Sstr3 and Mchr1 but is required for ciliary export of D1.

### Gpr161 and Gpr19 accumulate in cilia on Bbs1 knockout neurons

**FIGURE 3:**
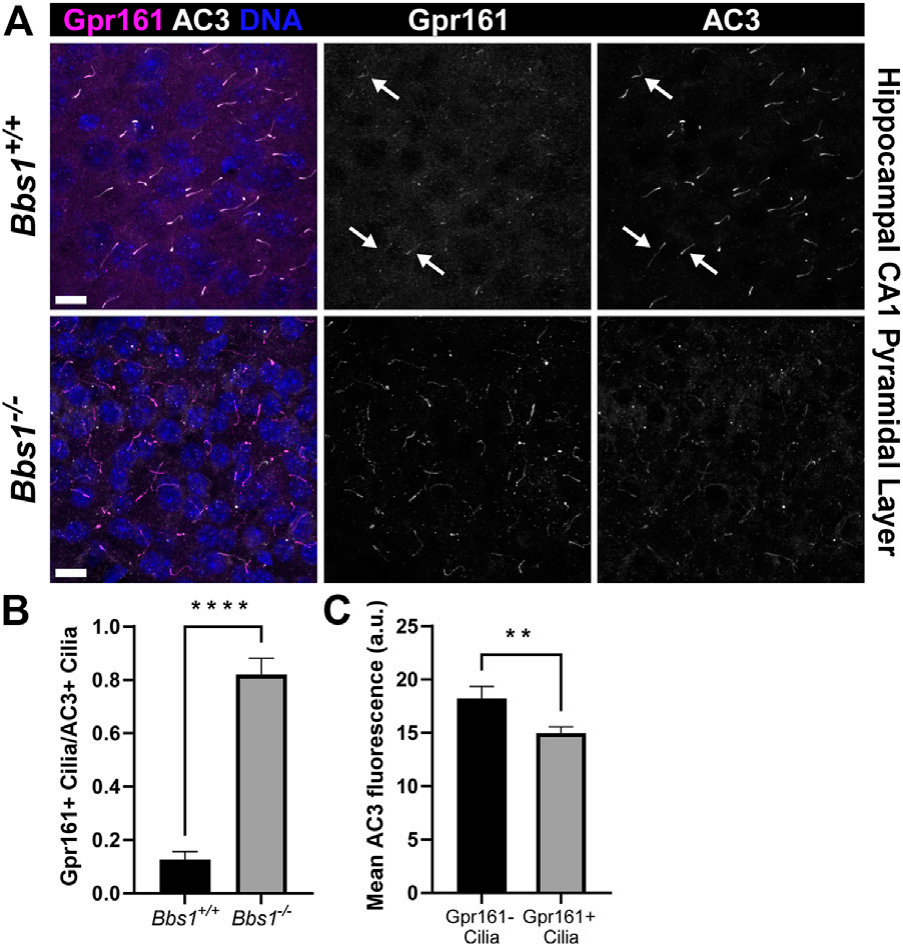
Gpr161 accumulates in cilia in the brains of *Bbs1* ^*−/−*^ mice and is associated with a reduction in AC3 ciliary signal **(A)** Representative images of the ventral CA1 region of the hippocampus from adult *Bbs1* ^*+/+*^ (upper row) and *Bbs1* ^*−/−*^ (lower row) mice colabeled for Gpr161 (magenta) and AC3 (white). Note that Gpr161-positive cilia are barely detectable in the *Bbs1* ^*+/+*^ section (indicated by arrows) but prominent in the *Bbs1* ^*−/−*^ section (middle panels). Nuclei are stained with DRAQ5 (blue). Scale bars represent 10 µm **(B)** Quantification of Gpr161-positive cilia relative to AC3-positive cilia reveals a significant increase in Gpr161-positive cilia in *Bbs1* ^*−/−*^ sections (889/1,093; *n* = 3 animals), compared to *Bbs1* ^*+/+*^ sections (91/959; *n* = 3 animals) **(C)** Quantification of mean ciliary AC3 fluorescence in Gpr161-negative (*n* = 74) and Gpr161-positive (*n* = 170) cilia in *Bbs1* ^*−/−*^ sections. Note that the AC3 signal is significantly lower in Gpr161-positive cilia. Values are expressed as mean ± SEM. **p < 0.01 ****p < 0.0001.

Gpr161 ciliary localization is restricted to pyramidal neurons in the CA1/CA2 regions of the adult mouse hippocampus (Mukhopadhyay et al., 2013). To test whether loss of Bbs1 impacts Gpr161 ciliary localization in the brain, we colabeled brain sections from adult *Bbs1* ^*+/+*^ and *Bbs1* ^*−/−*^ mice with antibodies against Gpr161 and AC3. In *Bbs1* ^*+/+*^ sections Gpr161-positive cilia were barely detectable (Figure 3A). However, in *Bbs1* ^*−/−*^ sections Gpr161-positive cilia were much more pronounced (Figure 3A), suggesting that the BBSome is required for Gpr161 ciliary export. Quantification of Gpr161-positive cilia in *Bbs1* ^*+/+*^ and *Bbs1* ^*−/−*^ sections confirmed that there are significantly more Gpr161-positive cilia in *Bbs1* ^*−/−*^ sections (t = 9.981, df = 27, *p* < 0.0001) (Figure 3B). Similar to D1 ciliary accumulation, we consistently observed a reduction in AC3 signal in Gpr161-positive cilia in *Bbs1* ^*−/−*^ sections. Quantification of mean ciliary AC3 fluorescence in Gpr161-negative and Gpr161-positive cilia in the hippocampal CA1 region of *Bbs1* ^*−/−*^ sections confirmed that AC3 signal is reduced in cilia that are enriched for Gpr161 (t = 2.779, df = 242, *p* < 0.01) (Figure 3C). Interestingly, quantification of the lengths of AC3-positive cilia in *Bbs1* ^*+/+*^ and *Bbs1* ^*−/−*^ sections revealed that cilia are significantly longer in *Bbs1* ^*−/−*^ sections (Table 1), which may contribute to the reduction in AC3 fluorescence.

**FIGURE 4:**
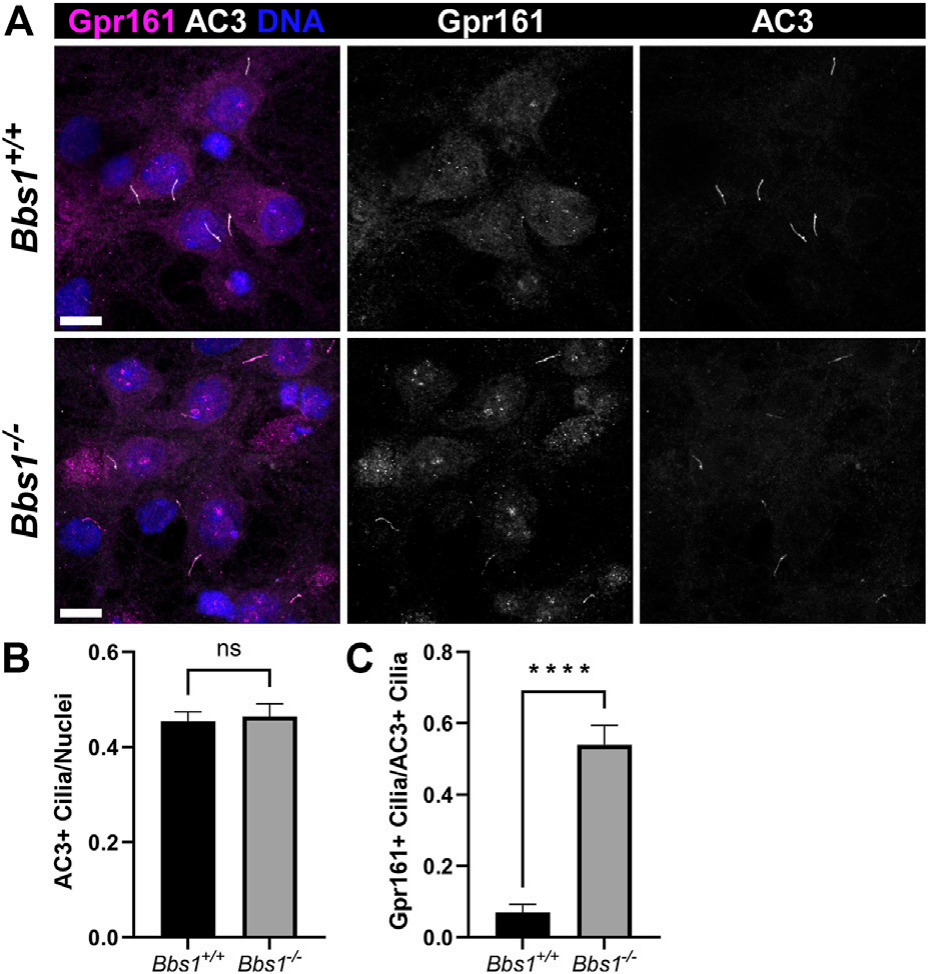
Gpr161 accumulates in cilia on *Bbs1* ^*−/−*^ hippocampal neurons **(A)** Representative images of cultured hippocampal neurons from *Bbs1* ^*+/+*^ (upper row) and *Bbs1* ^*−/−*^ (lower row) mice colabeled for Gpr161 (magenta) and AC3 (white). Note that Gpr161 ciliary localization is more pronounced in the *Bbs1* ^*−/−*^ culture (middle panels). Nuclei are stained with DRAQ5 (blue). Scale bars represent 10 µm **(B)** Quantification of AC3-positive cilia relative to nuclei reveals a similar fraction between *Bbs1* ^*+/+*^ (353/748; *n* = 3 animals) and *Bbs1* ^*−/−*^ (286/627 nuclei; *n* = 3 animals) cultures **(C)** Quantification of Gpr161-positive cilia relative to AC3-positive cilia reveals a significant increase in Gpr161-positive cilia in *Bbs1* ^*−/−*^ cultures (145/286; *n* = 3 animals), compared to *Bbs1* ^*+/+*^ cultures (18/353; *n* = 3 animals). Values are expressed as mean ± SEM. ns = not significantly different, ****p < 0.0001.

To confirm our *in vivo* findings, we colabeled cultured *Bbs1* ^*+/+*^ and *Bbs1* ^*−/−*^ hippocampal neurons with antibodies against Gpr161 and AC3. In *Bbs1* ^*+/+*^ cultures, we rarely observed Gpr161-positive cilia. The proportion of Gpr161-positive cilia we observed was significantly lower than what has been previously published (Badgandi et al., 2017) and may reflect differences in culturing and/or labeling procedures. Nevertheless, in *Bbs1* ^*−/−*^ cultures Gpr161-positive cilia were noticeably more abundant (Figure 4A). Quantification of AC3-positive cilia relative to nuclei in *Bbs1* ^*+/+*^ and *Bbs1* ^*−/−*^ cultures indicated that the proportion of cilia did not vary by genotype (t = 0.3262, df = 57, *p* = 0.74) (Figure 4B). However, quantification of Gpr161-positive cilia relative to AC3-positive cilia showed a significant increase in the proportion of Gpr161-positive cilia in *Bbs1* ^*−/−*^ cultures (t = 7.88, df = 57, *p* < 0.0001) (Figure 4C), providing further evidence that the BBSome is required for Gpr161 ciliary export. Our results are consistent with a previous study showing that Gpr161 accumulates in cilia on fibroblasts from mice homozygous for the M390R mutant *Bbs1* allele (Yee et al., 2015). Gpr161 also accumulates in cilia on BBSome mutant cell lines (Goetz et al., 2017; Nozaki et al., 2018).

**FIGURE 5:**
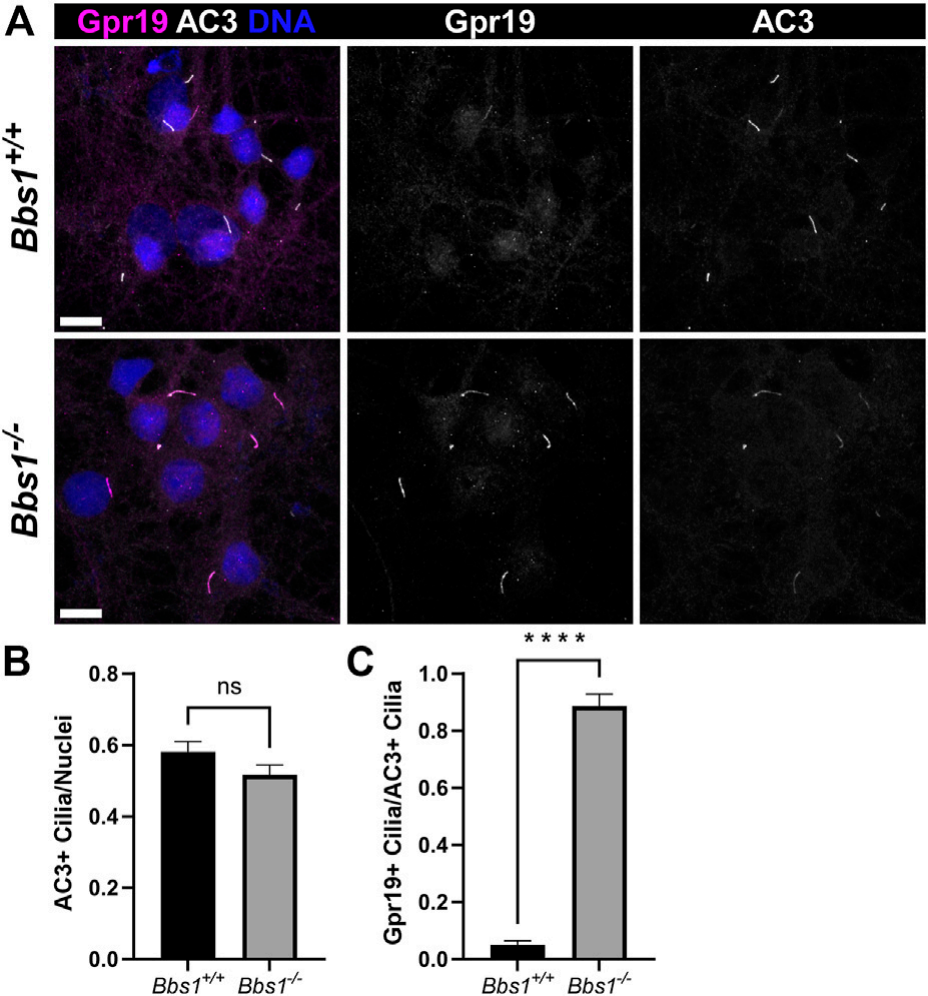
Gpr19 accumulates in cilia on *Bbs1* ^*−/−*^ hippocampal neurons **(A)** Representative images of cultured hippocampal neurons from *Bbs1* ^*+/+*^ (upper row) and *Bbs1* ^*−/−*^ (lower row) mice colabeled for Gpr19 (magenta) and AC3 (white). Note that Gpr19 ciliary localization is more pronounced in the *Bbs1* ^*−/−*^ culture (middle panels). Nuclei are stained with DRAQ5 (blue). Scale bars represent 10 µm **(B)** Quantification of AC3-positive cilia relative to nuclei reveals a similar fraction between *Bbs1* ^*+/+*^ (470/836; *n* = 3 animals) and *Bbs1* ^*−/−*^ (317/647; *n* = 3 animals) cultures **(C)** Quantification of Gpr19-positive cilia relative to AC3-positive cilia reveals a significant increase in Gpr19-positive cilia in *Bbs1* ^*−/−*^ cultures (270/317; *n* = 3 animals), compared to *Bbs1* ^*+/+*^ cultures (20/470; *n* = 3 animals). Values are expressed as mean ± SEM. ns = not significantly different, ****p < 0.0001.

Gpr19 is an orphan GPCR that also localizes to cilia on hippocampal neurons (Badgandi et al., 2017). As we were unable to detect Gpr19 in brain sections from adult *Bbs1* ^*+/+*^ and *Bbs1* ^*−/−*^ mice using our labeling procedures, we tested the impact of Bbs1 loss on Gpr19 ciliary localization by colabeling cultured *Bbs1* ^*+/+*^ and *Bbs1* ^*−/−*^ hippocampal neurons with antibodies against Gpr19 and AC3. Similar to Gpr161, we rarely observed Gpr19-positive cilia in *Bbs1* ^*+/+*^ cultures but observed abundant Gpr19-positive cilia in *Bbs1* ^*−/−*^ cultures (Figure 5A). Quantification of AC3-positive cilia, nuclei, and Gpr19-positive cilia revealed similar proportions of cilia for each genotype (t = 1.645, df = 58, *p* = 0.1) (Figure 5B) but a significant increase in the proportion of Gpr19-positive cilia in *Bbs1* ^*−/−*^ cultures (t = 18.68, df = 58, *p* < 0.0001) (Figure 5C). Thus, these results suggest the BBSome is also required for Gpr19 ciliary export.

### β-arrestin accumulates in subsets of cilia in the brains of *Bbs1* ^*−/−*^ mice

**FIGURE 6:**
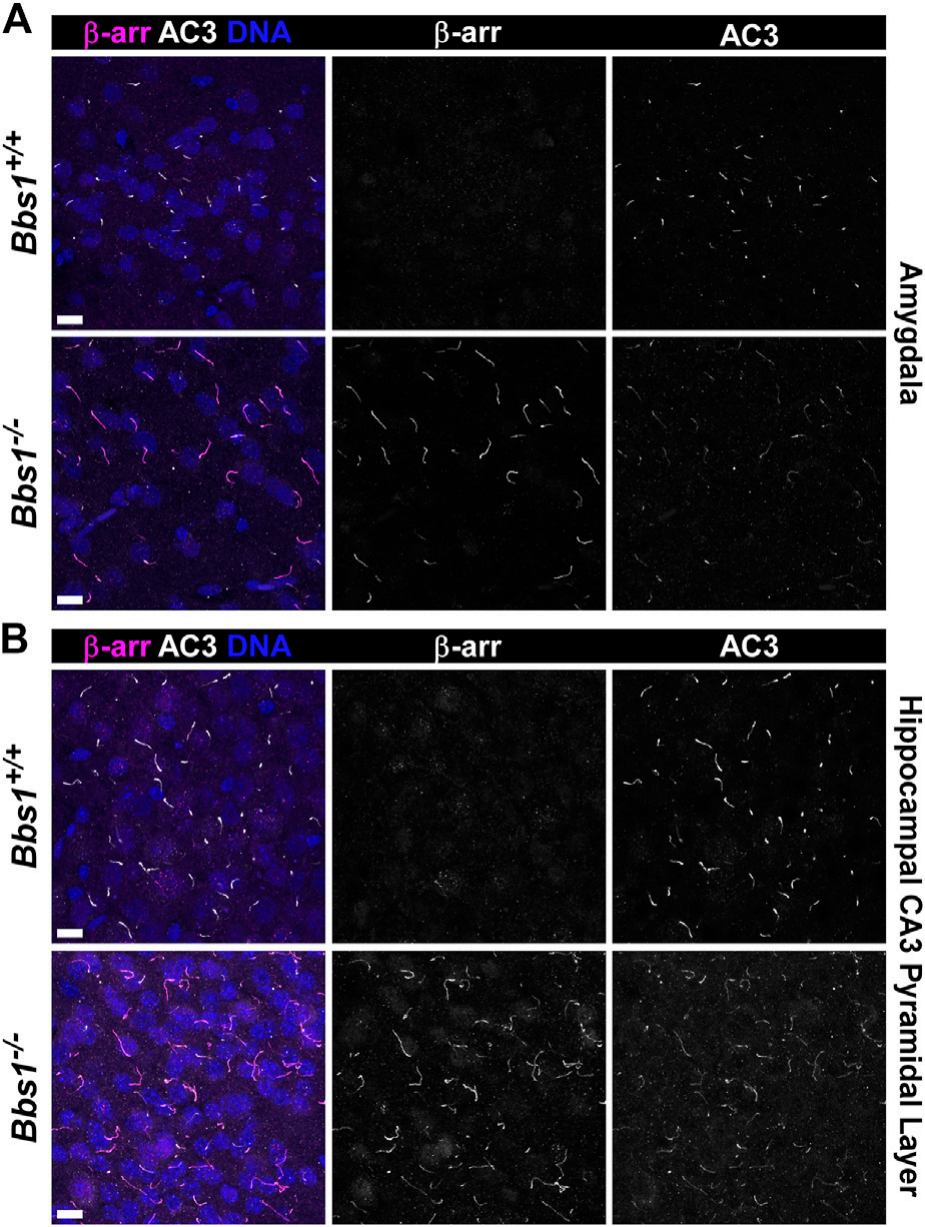
β-arrestin accumulates in cilia in the brains of *Bbs1* ^*−/−*^ mice **(A)** Representative images of the amygdala from adult *Bbs1* ^*+/+*^ (upper row) and *Bbs1* ^*−/−*^ (lower row) mice colabeled for β-arrestin (β-arr; magenta) and AC3 (white). β-arrestin localized to 0% of AC3-positive cilia in *Bbs1* ^*+/+*^ sections (0/1,540; *n* = 3 animals) and 90% of AC3-positive cilia in *Bbs1* ^*−/−*^ sections (712/790; *n* = 3 animals) **(B)** Representative images of the CA3 region of the hippocampus from adult *Bbs1* ^*+/+*^ (upper row) and *Bbs1* ^*−/−*^ (lower row) mice colabeled for β-arrestin (magenta) and AC3 (white). β-arrestin localized to 0% of AC3-positive cilia in *Bbs1* ^*+/+*^ sections (0/871; *n* = 3 animals) and 84% of AC3-positive cilia in *Bbs1* ^*−/−*^ sections (604/722; *n* = 3 animals). Nuclei are stained with DRAQ5 (blue). Scale bars represent 10 µm.

We previously showed that the GPCR signaling protein β-arrestin is recruited into neuronal cilia, ostensibly in response to ciliary receptor activation (Green et al., 2016). Further, β-arrestin 2 accumulates in cilia on a cell line lacking Bbs19 (Mick et al., 2015), suggesting that β-arrestin 2 requires BBS proteins for ciliary exit. To test whether loss of Bbs1 impacts β-arrestin localization to neuronal cilia, we colabeled brain sections from adult *Bbs1* ^*+/+*^ and *Bbs1* ^*−/−*^ mice with antibodies against β-arrestin and AC3. We did not observe β-arrestin-positive cilia in *Bbs1* ^*+/+*^ sections but observed abundant β-arrestin-positive cilia in *Bbs1* ^*−/−*^ sections (Figures 6A,B). β-arrestin-positive cilia were most abundant in the amygdala (Figure 6A) and on pyramidal neurons in the CA3 region of the hippocampus (Figure 6B) but were also found in numerous other brain regions, including the hypothalamus, thalamus, and cortex. β-arrestin did not accumulate in all cilia throughout the brain, suggesting that β-arrestin does not require the BBSome for ciliary exit or is only recruited into certain cilia.

**FIGURE 7:**
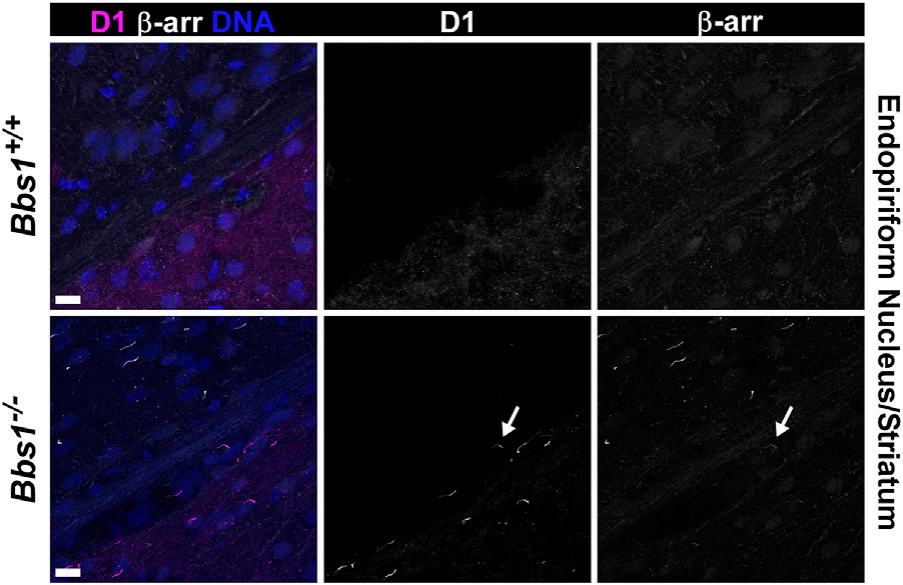
β-arrestin rarely accumulates in D1-positive cilia in the brains of *Bbs1* ^*−/−*^ mice. Representative images of the junction of the striatum (lower right corner) and the endopiriform nucleus (upper left corner) in adult *Bbs1* ^*+/+*^ (upper row) and *Bbs1* ^*−/−*^ (lower row) mice colabeled for D1 (magenta) and β-arrestin (β-arr; white). The striatum is distinguished by the presence of D1 labeling and the endopiriform nucleus is distinguished by a lack of D1 labeling. Note the lack of D1-or β-arrestin-positive cilia in the *Bbs1* ^*+/+*^ section but the presence of D1-positive cilia in the striatum and β-arrestin-positive cilia in the endopiriform nucleus of the *Bbs1* ^*−/−*^ section (left panels). A single cilium (indicated by an arrow) is positive for both D1 and β-arrestin (middle and right panel). β-arrestin colocalized to 0.9% (2/214; *n* = 3 animals) of D1-positive cilia in *Bbs1* ^*−/−*^ sections. Nuclei are stained with DRAQ5 (blue). Scale bars represent 10 µm.

To test whether D1 ciliary accumulation leads to β-arrestin ciliary accumulation, we colabeled brain sections from adult *Bbs1* ^*+/+*^ and *Bbs1* ^*−/−*^ mice with antibodies against D1 and β-arrestin. As expected, we did not observe D1-or β-arrestin-positive cilia in *Bbs1* ^*+/+*^ sections (Figure 7). In *Bbs1* ^*−/−*^ sections we observed abundant cilia that were positive for D1 or β-arrestin but D1 and β-arrestin colocalized in only 0.9% (2/214; *n* = 3 animals) of cilia (Figure 7). This result suggests that D1 ciliary accumulation is rarely associated with β-arrestin ciliary recruitment and accumulation.

## Discussion

Our results showing that Sstr3 and Mchr1 fail to localize to neuronal cilia in *Bbs1* ^*−/−*^ mice are consistent with a role for the BBSome in trafficking of some GPCRs into cilia. Other mechanisms have been implicated in the trafficking of GPCRs into cilia. The ubiquitously expressed tubby family protein Tulp3 directs ciliary localization of heterologous Sstr3, Mchr1, D1, Gpr161, and Gpr19 in cultured cell lines (Mukhopadhyay et al., 2010; Mukhopadhyay et al., 2013; Badgandi et al., 2017). Disruption of Tulp3 activity in cultured hippocampal neurons reduces Gpr161 and Gpr19 ciliary localization (Badgandi et al., 2017). Yet, neurons also express the founding tubby family member Tub. Interestingly, Tub is required for localization of Sstr3 and Mchr1 to neuronal cilia (Sun et al., 2012) but not Gpr161 and Gpr19 (Badgandi et al., 2017). Thus, it appears that Sstr3 and Mchr1 require Tub for ciliary localization whereas Gpr161 and Gpr19 can be trafficked to cilia by Tulp3. The basis for this differential requirement for GPCR ciliary localization in hippocampal neurons is unclear. It is interesting that GPCRs that are Tub-dependent fail to localize to cilia on BBSome mutant neurons and GPCRs that localize independently of Tub accumulate in cilia on BBSome mutant neurons. Perhaps, the BBSome and Tub act cooperatively to traffic a subset of GPCRs to cilia on neurons.

An alternative explanation for our results is that BBSome function is confined to ciliary GPCR export and Sstr3 and Mchr1 are constitutively ectocytosed from cilia on BBSome mutant neurons. Nager et al. (2017) investigated agonist-mediated ciliary export of heterologously-expressed Sstr3 in a ciliated renal epithelial cell line lacking BBSome or β-arrestin 2 function and found that activated Sstr3 is recruited to the ciliary tip and released in extracellular vesicles named ectosomes. A similar observation was made with NPY2 (Nager et al., 2017). Based on these findings it was hypothesized that the BBSome and β-arrestin 2 cooperatively export activated GPCRs from cilia and the loss of GPCR localization on neuronal cilia in BBSome mutant mice may be due to constitutive ectocytosis of the receptors rather than a defect in trafficking into cilia (Nager et al., 2017). Yet, we found that Gpr161 and Gpr19 accumulate in cilia on BBSome mutant hippocampal neurons, indicating that receptors can accumulate when ciliary export is blocked. Why would some ciliary receptors be constitutively ectocytosed while other receptors accumulate in cilia on the same population of neurons? One possibility is that Sstr3 and Mchr1 are activated on the ciliary membrane (a requirement for ectocytosis) but Gpr161 and Gpr19 are not. However, D1 ciliary localization decreases after agonist treatment in wildtype neurons, suggesting that D1 is activated on the ciliary membrane (Domire et al., 2011). Yet, in the absence of the BBSome D1 accumulates in neuronal cilia. An alternative possibility is that the BBSome traffics additional proteins into cilia that are required for activation of certain GPCRs. Nevertheless, our results show that the regulation of GPCR ciliary localization in neurons is complex and significant gaps remain in our understanding of GPCR trafficking into and out of cilia.

It is interesting to contemplate the consequences of Gpr161 ciliary accumulation in the brains of adult BBSome mutant mice. Shh signaling components, including Ptch and Smo, are expressed in rat adult hippocampal neurons (Petralia et al., 2011a; Petralia et al., 2011b). Shh treatment of hippocampal neurons regulates presynaptic terminal structure and function (Mitchell et al., 2012) and axon elongation (Yao et al., 2015), suggesting a role in synapse formation. Shh treatment also increases the density of dendritic spines on mature cultured mouse hippocampal neurons (Sasaki et al., 2010). As cilia are required for normal dendrite outgrowth of mouse cortical neurons (Guadiana et al., 2013), it is conceivable that cilia mediate Shh signaling on adult neurons. Interestingly, Bbs4 knockout mice have a significant reduction in dendritic spines on hippocampal neurons (Haq et al., 2019). It is possible that ciliary accumulation of Gpr161 on BBSome mutant hippocampal neurons disrupts Shh signaling and results in a reduction of dendritic spines. Smo accumulates in cilia of unstimulated BBSome mutant mouse embryonic fibroblasts (Zhang et al., 2012; Goetz et al., 2017), suggesting a role for the BBSome in the export of Smo from cilia. Thus, it is feasible that Smo and Gpr161 accumulate together in cilia on BBSome mutant hippocampal neurons, which could contribute to the increase in ciliary length. Further studies are necessary to test this possibility and determine the consequences for Shh pathway activity.

One model for GPCR ciliary export posits that upon receptor activation, β-arrestin is recruited into cilia where it facilitates receptor ubiquitination to mark its removal by the BBSome (Shinde et al., 2020). We detected β-arrestin accumulating in a subset of cilia throughout the brain of *Bbs1* ^*−/−*^ mice, suggesting these cilia contain an activated GPCR that requires the BBSome for ciliary export and upon recruitment β-arrestin binds to the activated receptor and becomes trapped in the cilium. Interestingly, β-arrestin was rarely observed in D1-enriched cilia. Possible explanations for this finding are that D1 is not activated on the ciliary membrane, β-arrestin is not recruited to activated D1, or the overaccumulation of D1 in the cilium prevents β-arrestin recruitment. β-arrestin is also recruited into cilia in response to Gpr161 activation (Pal et al., 2016; Shinde et al., 2020). Unfortunately, we were unable to colabel for β-arrestin and Gpr161 to test whether they colocalize within the same cilia since the antibodies are the same species. However, we observed Gpr161 ciliary accumulation in some brain regions where there was not β-arrestin ciliary accumulation. Thus, similar to D1, Gpr161 ciliary accumulation is not always associated with β-arrestin ciliary accumulation. It is likely that β-arrestin ciliary localization indicates the presence of additional known and unknown activated GPCRs on the ciliary membrane. An important area of future study will be identifying potential novel ciliary GPCRs and determining the causes of β-arrestin ciliary recruitment.

We previously reported a reduction in AC3 signal in D1-enriched cilia (Stubbs et al., 2022). In brain sections from *Bbs1* ^*−/−*^ mice we saw a significant decrease in AC3 signal in D1-and Gpr161-enriched cilia. Although we cannot rule out the possibility that D1 and Gpr161 ciliary accumulation interferes with binding of the AC3 antibody, it seems more likely that D1 or Gp161 accumulation prevents AC3 ciliary localization. It is tempting to speculate that GPCR overaccumulation in neuronal cilia crowds out other proteins, limits the availability of ciliary signaling partners, and disrupts GPCR ciliary signaling. Functional studies utilizing live-cell imaging and genetically-encoded signaling reporters should reveal whether AC3-mediated cAMP signaling is impacted by GPCR ciliary accumulation.

In summary, we have shown that Sstr3 and Mchr1 fail to localize to cilia, while D1, Gpr161, and Gpr19 accumulate in cilia on neurons from *Bbs1* ^*−/−*^ mice. We further show that β-arrestin accumulates in a subset of cilia throughout the brain of *Bbs1* ^*−/−*^ mice but is rarely found in D1-enriched cilia. Our findings provide additional support for a role for BBS proteins in the export of ciliary GPCRs in the brain but raise questions about why some GPCRs are absent from cilia and others accumulate in cilia.

## Materials and methods

### Mice and tissue preparation

All mouse studies were conducted in accordance with institutional guidelines based on National Institutes of Health standards and were performed with Institutional Animal Care and Use Committee approval at the Ohio State University. All animals were maintained in a temperature and humidity-controlled vivarium with 12-h light/dark cycle at a maximum of five per cage and given access to normal chow and water *ad libitum*. Generation of the germline Bbs1 deleted (null) allele has been previously described (Stubbs et al., 2022). To increase the viability of Bbs1-null animals, *Bbs1* ^*wt/null*^ mice on a congenic background (C57BL/6J) were crossed with FVB/NJ mice from the Jackson Laboratory (#001800). Constitutive *Bbs1* ^*null/null*^ mice were generated by intercrossing *Bbs1* ^*wt/null*^ male and female mice on a mixed background. Fixed mouse brains were generated and processed as previously described (Berbari et al., 2008b), and sliced at a thickness of 20 µm. Neurons were cultured as previously described (Green et al., 2016), with the exception that neurons were cultured in Neurobasal plus medium containing B-27 plus supplement (Thermo-Fisher).

### Immunofluorescence

Seven to 10 days after plating, neuronal cultures were fixed and processed for immunofluorescence as previously described (Green et al., 2016). Primary antibodies included anti-AC3 (mouse, EnCor MCA-1A12 1:1,000; rabbit, EnCor RPCA-ACIII 1:1,000, chicken, EnCor CPCA-ACIII 1:1,000) (Wong et al., 2000), anti-Sstr3 (goat, Santa Cruz M-18 1:250; rabbit, Gramsch ss-830 1:100) (Handel et al., 1999), anti-Mchr1 (rabbit, Invitrogen 711,649 1:250) (Jasso et al., 2021), anti-D1 (mouse, Santa Cruz sc-33660 1:500) (Domire et al., 2011), anti-pan-arrestin (rabbit, Abcam Ab2914 1:500) (Jones and Hinkle, 2008). Anti-Gpr161 (rabbit, 1:250) (Mukhopadhyay et al., 2013) and anti-Gpr19 (rabbit, 1:250) (Badgandi et al., 2017) were a kind gift from Saikat Mukhopadhyay (UT Southwestern). Secondary antibodies included Alexa fluor 488- and 546-conjugated donkey anti-mouse, Alexa fluor 488- and 546-conjugated donkey anti-rabbit, Alexa fluor 488-conjugated donkey anti-goat, and Alexa fluor 546-conjugated goat anti-chicken (Invitrogen 1:1,000). Nucleic acids were stained with DRAQ5 (Thermo-Fisher Scientific, 1:5,000). Samples were imaged on a Leica TCS SP8 laser scanning confocal microscope at the Hunt-Curtis Imaging Facility in the Department of Neuroscience at OSU. Multiple consecutive focal planes (Z-stack), spaced at 0.3–0.5 μm intervals, were captured.

### Quantification and statistical analysis of neuronal cilia

Quantification of cilia in brain slices was performed using coronal sections from 5–7-week-old *Bbs1* ^*+/+*^ and *Bbs1* ^*−/−*^ littermates (2 male and one female of each genotype). For each section, six to nine fields were imaged and the number of Sstr3-, Mchr1-, D1-, Gpr161-, arrestin-, and AC3-positive cilia was counted by an individual blind to the genotype. The results were expressed as the proportion of AC3-positive cilia that were positive for each GPCR or arrestin. To quantify relative ciliary AC3 levels, fluorescence intensity of ciliary AC3 in D1-positive and -negative cilia and Gpr161-positive and -negative cilia were measured in ImageJ. ROIs were drawn around individual cilia and the mean fluorescence intensity of the cilium compared to background was calculated. The fluorescence intensity of AC3 in D1-and Gpr161-positive cilia were averaged and compared to the average fluorescence intensity of AC3 in D1-and Gpr161-negative cilia, respectively. Quantification of ciliary lengths was measured in ImageJ by drawing lines calibrated to the scale bar along the length of AC3-positive cilia. Quantification of Gpr161-and Gpr19-positive cilia in cultured neurons was performed on three coverslips from three animals for each condition and genotype. For each coverslip at least six fields were imaged. The number of nuclei, Gpr161-or Gpr19-positive cilia, and AC3-positive cilia was counted by an individual blind to the genotype. The results were expressed as the proportion of AC3-positive cilia to nuclei and the proportion of Gpr161-or Gpr19-positive cilia to AC3-positive cilia. Data were analyzed using Prism nine software (GraphPad Software) and results were expressed as mean ± SEM. Statistical significance was tested using Student’s *t*-test.

## Data Availability

The raw data supporting the conclusions of this article will be made available by the authors, without undue reservation.
