# Supplementary material for: Ciliary signaling proteins are mislocalized in the brains of Bardet-Biedl syndrome 1-null mice
Source: Front Cell Dev Biol. 2023 Jan 9;10:1092161. doi: 10.3389/fcell.2022.1092161 (PMC9868275; doi:10.3389/fcell.2022.1092161)
Supplement: Supplementary file 1 [file Table1.DOCX]

| Region | GPCR | *Bbs1^+/+^*  localization | *Bbs1^-/-^*localization | Cilia lengths (µm)^a^ |
| --- | --- | --- | --- | --- |
| CA3 region hippocampus^b^ | Sstr3 | Y | N | *Bbs1^+/+^*: 4.203 ±0.08123  *Bbs1^-/-^*: 3.991 ±0.1034  **Not significantly different**  p=0.1060 |
| Nucleus accumbens^c^ | Mchr1 | Y | N | *Bbs1^+/+^*: 5.825 ±0.1160  *Bbs1^-/-^*: 5.995 ±0.1808  **Not significantly different**  p=0.4069 |
| Striatum^d^ | D1 | N | Y | *Bbs1^+/+^*: 6.625 ± 0.1931  *Bbs1^-/-^*: 7.179 ± 0.3075  **Not significantly different**  p=0.1243 |
| Ventral CA1 region^e^ | Gpr161 | Y | Y | *Bbs1^+/+^*: 4.520 ± 0.1151  *Bbs1^-/-^*: 5.424 ± 0.2135  **Significantly different**  p<0.0001 |

**Table 1: Summary of GPCR ciliary localization and ciliary length in *Bbs1^+/+^* and *Bbs1^-/-^* brain regions.**

^a^ Lengths recorded as mean (µm) ± S.E.M.

^b^ In the CA3 region of the hippocampus n = 390 *Bbs1^+/+^* cilia and n = 379 *Bbs1^-/-^* cilia

^c^ In the nucleus accumbens n = 390 *Bbs1^+/+^* cilia and n = 237 *Bbs1^-/-^* cilia.

^d^ In the striatum n = 135 *Bbs1^+/+^* cilia and n = 128 *Bbs1^-/-^* cilia.

^e^ In the ventral CA1 region of the hippocampus n = 258 *Bbs1^+/+^* cilia and n = 190 *Bbs1^-/-^* cilia.
